# Prognostic value of dual-source computed tomography (DSCT) angiography characteristics in anomalous coronary artery from the opposite sinus (ACAOS) patients: a large-scale retrospective study

**DOI:** 10.1186/s12872-019-01285-3

**Published:** 2020-01-17

**Authors:** Kai-yue Diao, Qin Zhao, Yue Gao, Ke Shi, Min Ma, Hua-yan Xu, Ying-kun Guo, Zhi-gang Yang

**Affiliations:** 1 Department of Radiology, State Key Laboratory of Biotherapy West China Hospital, Sichuan University, No.37 Guoxue Xiang, Chengdu, 610041 China; 2 Department of Cardiology, West China Hospital, Sichuan University, Chengdu, China; 3 Department of Radiology, Key Laboratory of Birth Defects and Related Diseases of Women and Children of Ministry of Education, West China Second University Hospital, Sichuan University, 20# Section 3 South Renmin Road, Chengdu, 610041 China

**Keywords:** Coronary vessel anomalies, Computed tomography angiography, Congenital abnormalities, Diagnosis, Prognostic factor

## Abstract

**Background:**

Most reported cases of right anomalous coronary artery from the opposite sinus (R-ACAOS) have benign clinical outcomes. However, patients with left ACAOS (L-ACAOS) and some of the patients with R-ACAOS are more at risk for arrhythmias and sudden cardiac death, which remains a major concern. Here we report the prevalence and anatomical features of ACAOS patients. Moreover, we explore the high-risk morphological signs and evaluate their mid-term prognostic value in R-ACAOS patients without surgical intervention.

**Methods:**

Data from coronary computed tomography angiography (CTA) of 30,593 patients, pertaining to a single center over 5 consecutive years, were retrospectively analyzed. The image analysis included stenosis severity ranking and high-risk anatomy evaluation, based on the commercially available image post-processing software OsirX. Patients with R-ACAOS and without evidence of coronary atherosclerosis (CAD) were followed-up, with recording of the cardiovascular clinical events. Cox regression analysis was performed to identify the potential anatomical risk factors of cardiovascular clinical events for non-CAD R-ACAOS patients, using R project.

**Results:**

The prevalence of ACAOS in the study population was 0.69% (211/30593). Significant differences were found between patients with mild (< 50%) and severe (> 50%) stenosis, in terms of height-to-weight ratio (HW ratio), take-off angle, and proximal stenosis length. A total of 54 cardiovascular clinical events were observed among 108 non-CAD R-ACAOS patients and an average follow-up of 27.8 ± 18.7 months. Among those patients’ anatomical features, stenosis severity was the main risk factor for cardiovascular clinical events during the mid-term follow-up, with a risk ratio of 4.14 (95% CI: 1.78 to 9.63, *P* < 0.001).

**Conclusions:**

Among patients referred to coronary CTA, the overall incidence of ACAOS was 0.69%. For patients with R-ACAOS, severe stenosis was the independent risk factor of adverse clinical events in the mid-term follow-up, and positive clinical intervention might be needed to help them avoid the malignant clinical events.

## Background

Anomalous coronary artery from the opposite sinus (ACAOS) has long been recognized as a common type of congenital coronary artery malformation [1] with a prevalence between 0.14 and 1.7%. Although this anomaly has been considered as relatively “friendly,” compared with other congenital diseases, epidemiological studies have identified left ACAOS (L-ACAOS) and some of the right ACAOS (R-ACAOS) as a major factor of sudden cardiac death (SCD) in young adults [2], especially competitive athletes [3], and a number of patients with this anatomical variation present similar symptoms to those with coronary artery disease (CAD). Although vascular surgical intervention for vessel route reconstruction has been shown to be highly safe [4, 5], its necessity and indications remain controversial [6]. Recent studies have demonstrated associated high-risk anatomical variations and suggest them as screening items for special working groups involved with intensive myocardial exercise [1].

Coronary computed tomography angiography (CTA), especially advanced multi-slice computed tomography and dual-source computed tomography (DSCT), provides a noninvasive way to obtain a dedicated overview of coronary arteries with high temporal and spatial resolution. Its diagnostic value for multiple coronary artery anomalies and for critical surgical indications has been widely reported over the past 10 years [7]. Other recommended methods for plaque characterization and observation of coronary ostium anomalies include transesophageal echocardiography (TEE) [8] and intravascular ultrasonography [9]. However, these are inevitably limited by a relatively lower capacity for anatomical description and dependence on operating physicians. Although magnetic resonance imaging is also proposed when physiologic details are required, it is inferior to coronary CTA for coronary artery morphological evaluation [8].

A review of previous reports on ACAOS revealed a lack of simultaneously documented high-risk anatomical features and follow-up in a large sample of ACAOS patients without surgery. A prominent data deficiency from China is noted (Table 1). Moreover, in some cases (when the anomalous left main or left anterior descending artery coursing between the aorta and pulmonary artery) where L-ACAOS is considered malignant and more emphasis or surgical therapy is provided to the patients, treatment for patients with R-ACAOS has not been completely decided, and the prognosis of such patients with conservative therapy is also encouraged [12]. Usually our center does not perform anatomy correction surgery in such patients unless absolutely necessary or if the patients strongly require it. To fill these gaps, we retrospectively collected CT angiography data from our center pertaining to the past 5 years, analyzed their anatomical characteristics, and tracked the R-ACAOS natural history, aiming to demonstrate the epidemiological and anatomical features of R-ACAOS in our area and to recognize the potential high-risk CTA characteristics to predict a relatively poor prognosis.

**Table 1:** Overview of the ACAOS studies published from 2010

Study	Area, Study-time	Prevalence(N)	Age	R/L	Focus
Frommelt PC, 2011 [10]	America, 1999–2009	NA (27)	55.3 ± 13.5	20/7	B
Mumtaz MA, 2011 [11]	America, 1998–2008	NA (22)	15(5–54) ^a^	15/7	B
Krasuski, RA, 2011 [12]	America, 1966–2007	0.14%(301/210700)	57.7 ± 14.3	238/63	B
Xu,H, 2011 [13]	Asia, 2006–2011	0.57%(69/12145)	57.6^a^	51/18	C
Miller JA, 2012 [14]	America, 2004–2012	NA (15)	37.3 ± 18.9	10/5	A
Lee HJ, 2012 [15]	Asia, 2005–1020	0.68%(156/22925)	56^a^	124/32	A, C
Tuo G, 2013 [16]	European, 1994–2006	0.55%(44/7960)	67 ± 7.2	14/30	C
Opolski MP, 2013 [17]	European, 2008–2012	0.84(72/8522)	55 ± 12	20/53	A, C
Krupiński M, 2014 [18]	European, 6 consecutive years	0.76% (54/7115)	60.9 ± 11.6	16/38	A
Mainwaring RD, 2014	America,1999–2013	NA (69)	15^b^	47/22	B
Sharma V, 2014 [19]	America,1992–2011	NA (75)	39.6 ± 19.6	69/6	B
Poynter JA,2014 [20]	America,1998–2009	NA (196)	10.2^a^	144/51	B
Ashrafpoor G. 2015 [21]	European,2008–2013	0.46%(19/4160)	47.05 ± 9.75	16/38	A
Nasis A, 2015 [22]	Australia,2008–2013	1.09%(107/9774)	58.1 ± 12.6	36/71	A, C
Kooij M, 2015 [23]	European,201–2014	NA (31)	38^a^	26/5	B
Feins EN, 2015 [6]	America,1974–2014	NA (259)	42.5 ± 2.7	24/6/1	B
Cheezum MK, 2016 [24]	America,2004–2014	2.2% (131/5991)	52 ± 17	45/63	A
Law Timothy, 2016 [5]	Australia,2002–2014	NA (16)	46.6 ± 16.1	16/0	B
Driesen BW, 2018 [25]	Netherlands, 2010–2017	NA	52 (36–64)	25/5	C

A: High-risk anatomy; B: surgical intervention; C: follow-up prognosis; N: number of patients

^a^The standard deviation was not applicable

^b^The value is expressed in median with 0.25 to 0.75 quantile

## Methods

### Patient selection

Coronary CTA data from a total of 30,593 consecutive patients were initially reviewed, including those scheduled for DSCT from January 2012 to February 2017 at a single medical center, either because of CAD suspicion or physical examinations. Written informed consent was obtained from the studied group before CTA, including a notice of radiation exposure and associated adverse reactions to the contrast agent, prior to CTA scan. Any personal patient information was treated appropriately, and the follow-up data was safely saved.

Data were discarded if the patients (1) had congenital heart diseases likely to cause severe hemodynamic disorders, other than ACAOS and complex vascular abnormalities, such as transposition of the great artery; (2) underwent any vascular reconstruction surgeries before the CCTA; or (3) had other high-risk coronary artery abnormalities, such as anomalous origin of coronary arteries from the pulmonary trunk, coronary artery atresia, coronary artery fistula, and coronary aneurysms.

### Coronary CTA

Scanning was performed using a retrospective electrocardiography-gated protocol and the shared tube voltage and effective tube current for DSCT ((Somatom Definition Flash; Siemens Medical Solutions, Forchheim, Germany) were 100–120 kV (adapted to body mass index) and 220 mAs respectively. A non-ionic contrast agent (iopamidol, 370 mg/ml, Bracco, Milan, Italy) was injected at a flow rate of 5 ml/s and followed by 20 ml of saline solution for each patient. The final images were reconstructed using a slice thickness of 0·75 mm and an increment of 0·70 mm.

### Image analysis

Post-processing of the acquired coronary CTA images was performed on a clinically routine workstation (Syngo.via; Siemens Medical System, Forchheim, Germany) and OsiriX (Pixmeo; Switzerland; version 4.0). Reconstructed images parallel and perpendicular to the long-axis of the proximal course of the anomalous artery and curved multi-planar images were used to determine the presence of ACAOS and evaluate the related morphological features. The dominant coronary artery and the major accompanying congenital anatomic variations or abnormalities were recorded including myocardial bridging, an abnormal number of coronary arteries, hypoplastic or hypertrophic coronary artery and accessory right coronary arteries, as well as atherosclerosis and the stenosis degree of the coronary arteries. Patients were divided into CAD and non-CAD group by the presence of atherosclerosis in any of the coronary arteries to compare the anatomic features. And the patients with atherosclerotic plaques were further classified and referred for downstream investigations or cardiologists following the newest CAD-RIDS guidelines [26].

**Fig. 1:**
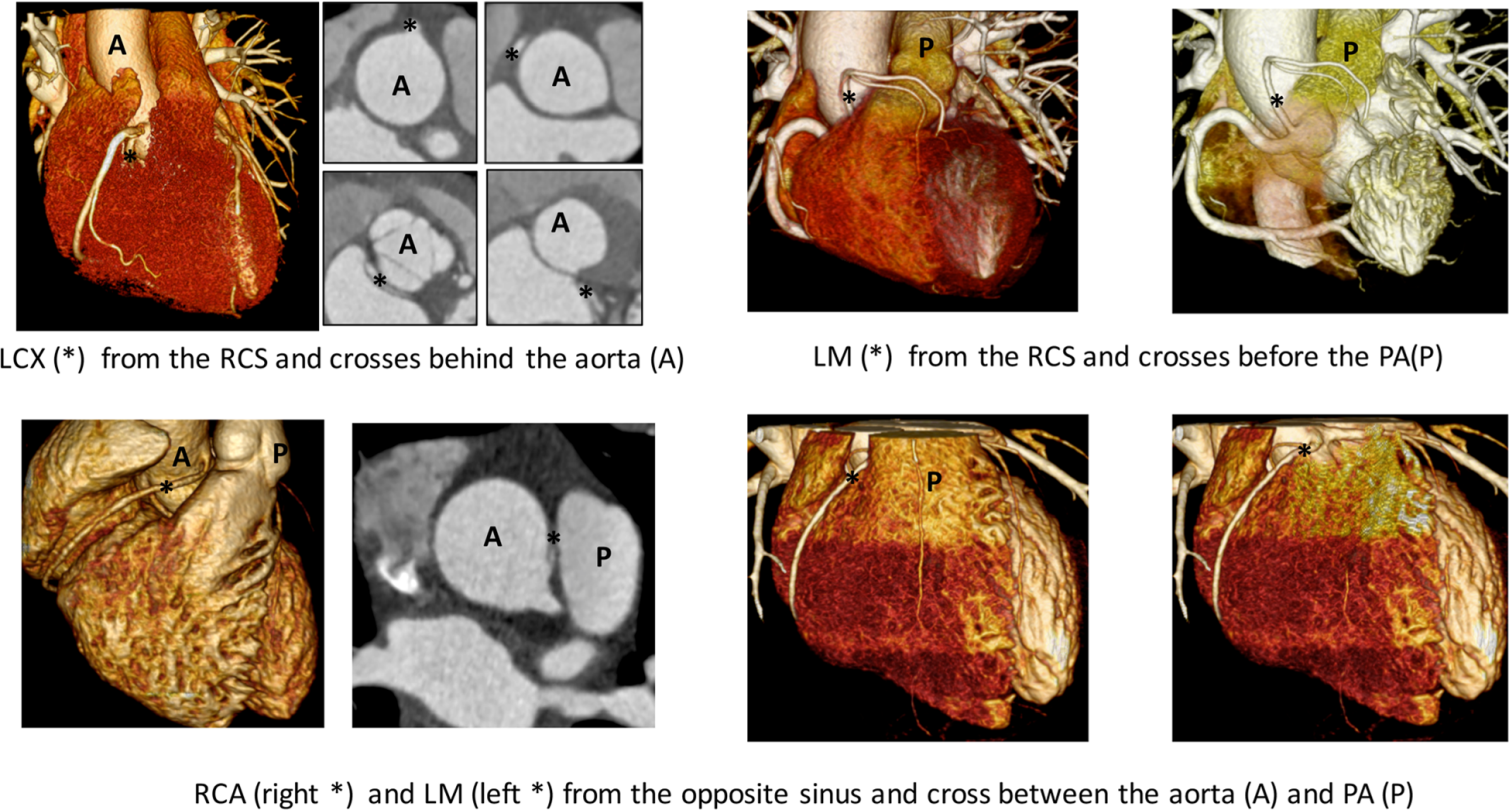
Three-dimensional reconstruction images and the corresponding axial view of the abnormal coronary artery (*) for LCX-retroaortic (upper-left), LM-prepulmonic (upper-right), RCA-parapulmonic (lower-left), and LM-interarterial (lower-right). LCX: left circumflex; LM: left main branch; RCA: right coronary artery; PA: pulmonary artery; A: aorta

**Fig. 2:**
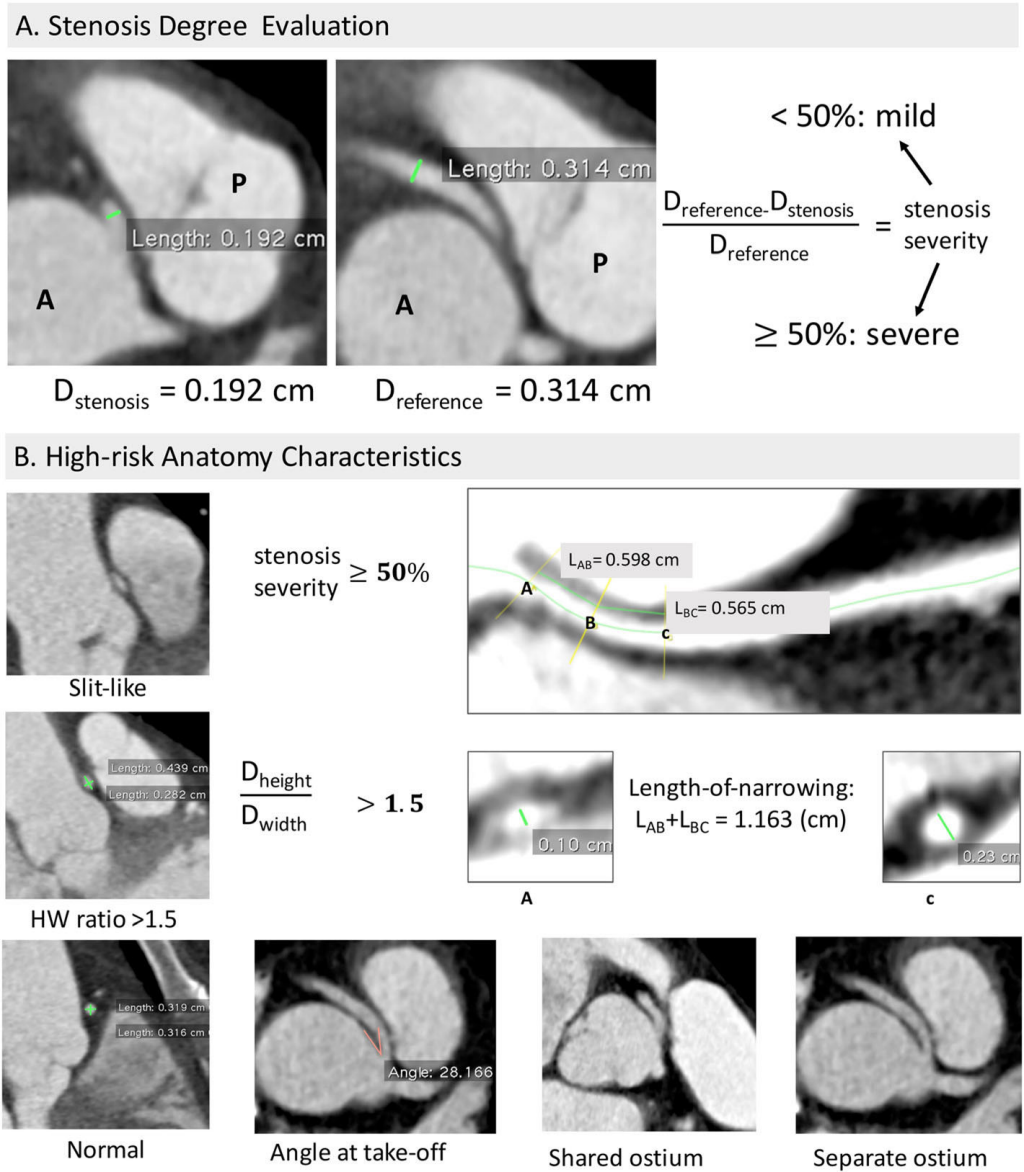
CTA image analysis protocol for the ACAOS. **a** For stenosis severity ranking, a degree of stenosis less than 50% were classified as mild stenosis and a degree of stenosis equal or more than 50% were classified as severe stenosis. **b** the high-risk anatomy characteristics identification protocol, including “slit-like” vessel, Height-to-width ratio (HW ratio), angle at the take-off level, and the shared or separate ostium

The basic anatomic assessment of the abnormal coronary branch consisted of measurement of the diameters at the most narrowed site at the end of the diastole phase, proximal stenosis length [18], take-off angle [27], characteristic ostium (separate or sharing with the contralateral branch), and the relationship with the surrounding structures (retroaortic, prepulmonic, subpulmonic, parapulmonic, suprapulmonic or interarterial) (Fig. 1). Furthermore, the diameters of the analysed coronary artery at the normal sites, as well as that of other main coronary branches were taken as reference. The degree of stenosis was determined by the diameter ratio of the most narrowed site to the corresponding normal reference (Fig. 2a). The take-off angle and height-to-width ratio (HW ratio) at the cross-section of the anomalous vessel [14] were also measured accordingly and shape of the anomalous artery was decided based on HW ratio according to the study by Agrawal H, et al. [28] (Fig. 2b).

One trained cardiologist and 2 training fellows, who were blinded to the clinical data, separately performed the image analysis. Any disagreement was resolved by another radiologist with 30 years of imaging working experience. Anatomical land markers and segments were referred to the modified American Heart Association’s 16-segment coronary artery model.

### Clinical information

Typical angina complaints including chest pain, syncope, dyspnea, and palpitation were recorded, as well as baseline characteristics, including age, sex, examination date, symptoms, and other complications. Telephone follow-up was performed (by G.Y) for R-ACAOS patients without prominent plaques on the coronary CTA images. Relevant cardiovascular clinical events were recorded if the patient (1) had typical anginal complaints or acute coronary syndromes; (2) underwent clinical intervention for the ACAOS for frequent angina symptoms, which should be suggested by the cardiac experts after carefully reviewing and systematically assessing the anatomy of the patients and recorded as medication, percutaneous coronary intervention, CABG, coronary unroofing, or reimplantation; (3) had malignant arrhythmia diagnosed by cardiac experts, including ventricular tachycardia or ventricular fibrillation; (4) or had an acute death without other specific clinical diseases. Major adverse clinical events (MACE) included acute death, acute coronary syndrome, ACAOS revascularization surgery, or malignant arrhythmia.

### Radiation dose estimation

Measurements of the volume CT dose index (CTDI) and dose-length product (DLP) were collected on the basis of the individual dose report. The DLP was converted to the effective dose (ED) as suggested by the previously published guidelines [23, 29].

### Statistical analysis

Continuous variables were presented as the mean ± standard deviation and compared using Student’s t-test and the Wilcoxon test for 2 different independent groups.

Categorical variables were expressed as frequency and compared using the chi-square test. Linear regression was used for analysing the correlations between stenosis severity and the high-risk factors when prominent differences between the two groups with different severity levels were observed. A generalised model was utilised to study the correlations between the significant high-risk factors and the patients’ follow-up data. ROC analysis and Cox analysis were further performed to determine the best threshold for high-risk anatomy characteristics and relative risk ratio (RR) to predict the relevant cardiovascular events. R project software (version 3.3.1) was used to perform the statistical analysis. A *P* value < 0·05 was considered significant.

## Results

### Baseline characteristics

**Fig. 3:**
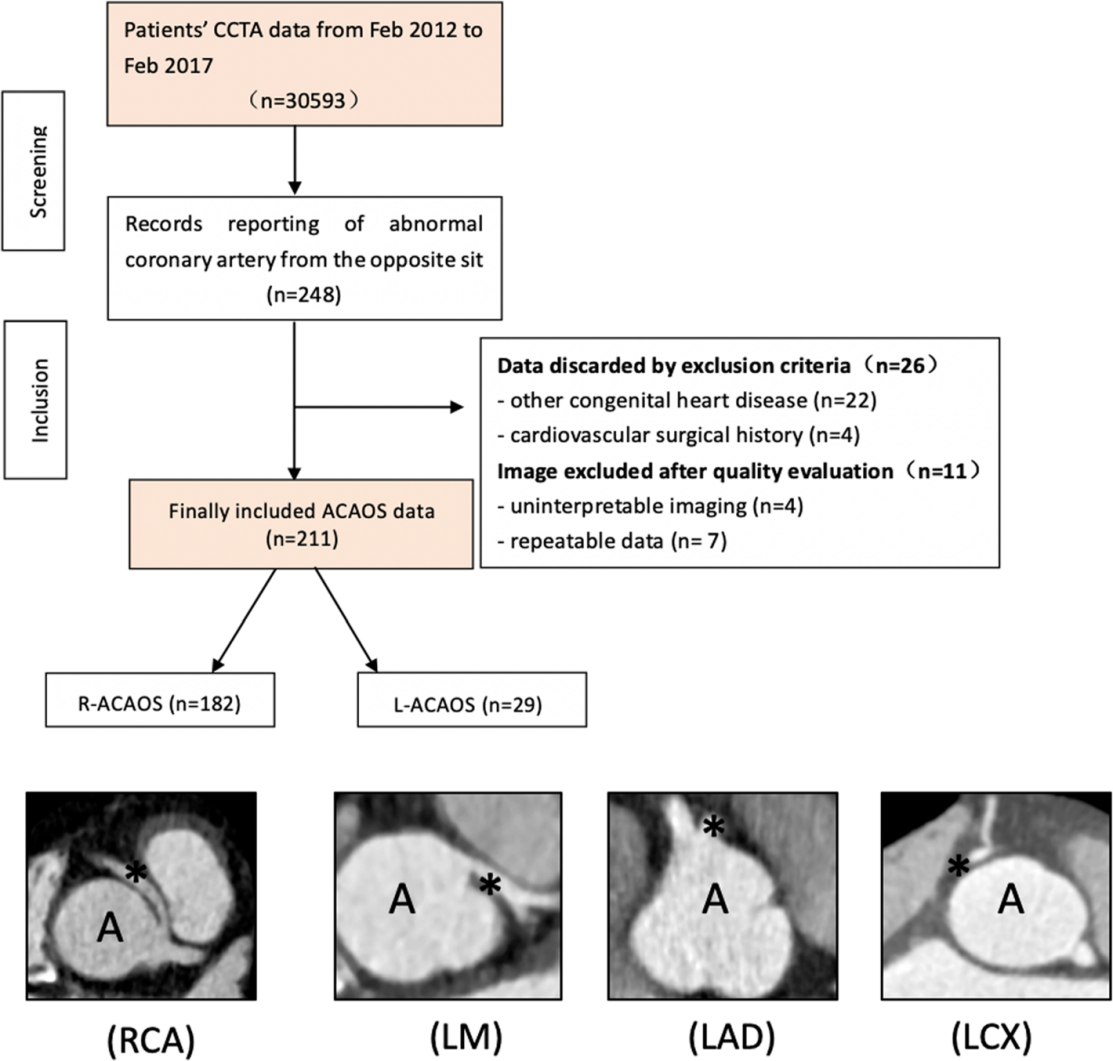
The Flow chart of the study. CCTA: coronary computed tomography angiography; ACAOS: anomalous coronary artery from the opposite Sinus; RCA: right coronary artery; LM: left main branch; LAD: left anterior descending branch; LCX: left circumflex branch

**Fig. 4:**
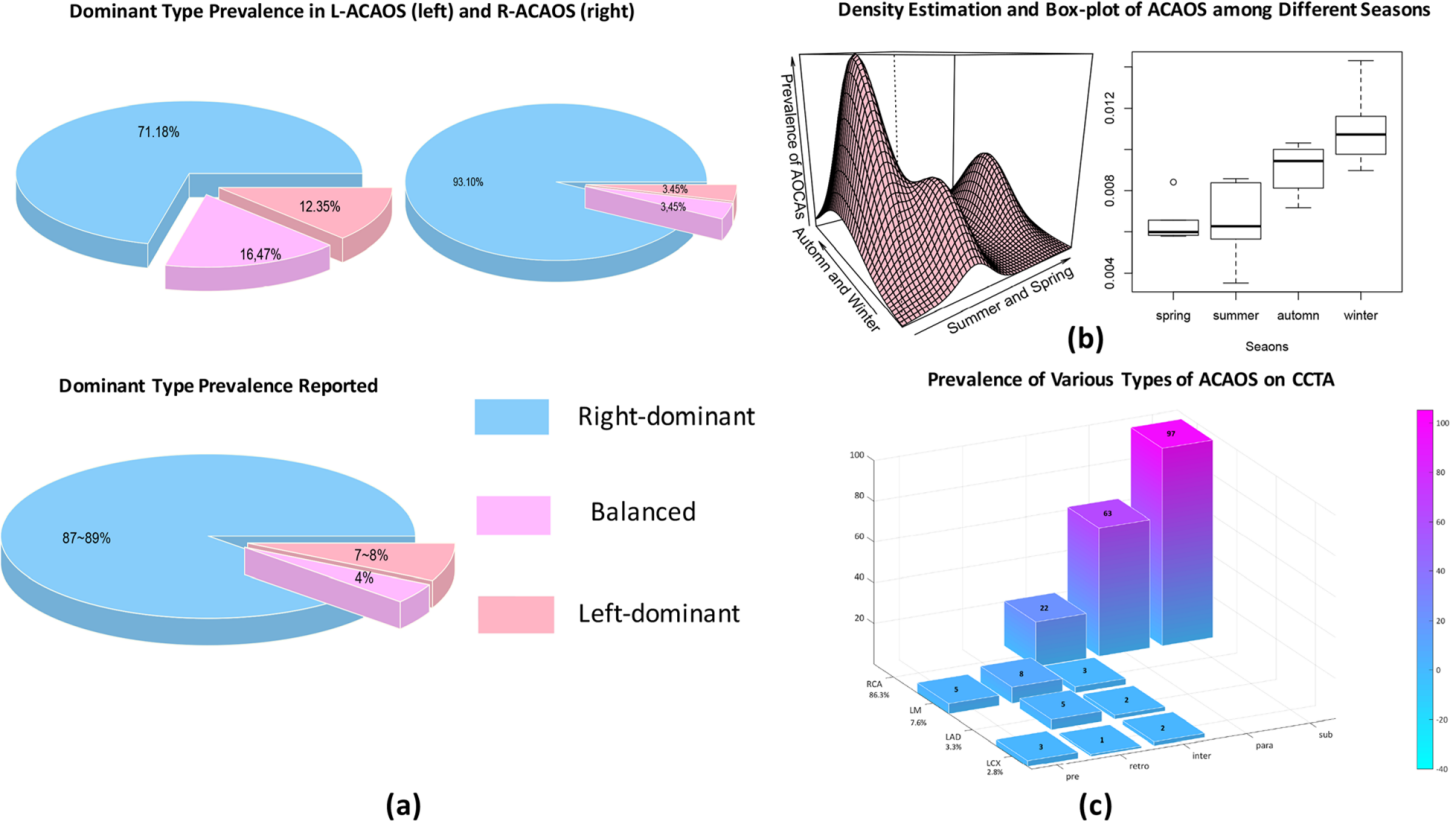
The overall distribution of ACAOS among different seasons and types. **a** The percentage of different dominant coronary type comparison between the reported coronary CTA findings and ACAOS. **b** The density estimation and box plot reveal a visual difference of ACAOS prevalence in different seasons throughout the years. **c** The percentages of ACAOS subtypes on coronary CTA. The subpulmonic type (*n* = 97, 53·3%) was the most common in R-ACAOS, while the retroaortic type (*n* = 14, 48·3%%) was the most common in L-ACAOS

Two hundred and forty-eight patients were screened for the initial study population. After excluding 22 for other congenital cardiac anomalies, 4 for a history of cardiovascular reconstruction surgeries, 4 for uninterpretable imaging, and 7 for repeatable data, data from 211 patients (prevalence of 0.69% on coronary CTA) finally remained (Fig. 3). Among these, 182 were R-ACAOS and 29 were L-ACAOS. The average radiation (DLP) and effective doses were 30.98 ± 17.96 mGy-cm and 4.43 ± 2.54 mSv, respectively, remaining within the suggested range [28]. A major seasonal tendency of confirmed ACAOS patients was noted, indicating that the occurrence of ACAOS patients was higher during the winter than during other seasons (1.2, 0.7, 0.6, and 0.3% for winter, autumn, spring, and summer, respectively) (Fig. 4b).

ACAOS patients were divided into 2 groups, CAD (*n* = 82, age: 64.5 ± 12.2) and non-CAD (*n* = 129, age: 55.3 ± 13.5), according to the presence of atherosclerosis plaques. Hypertension (38.2%), diabetes mellitus (24.5%), and hyperlipidemia (29.2%) were the most common cardiovascular comorbidities, and no significant differences were found among the groups. Chest pain was the most frequent complaint in patients referred for coronary CTA (Table 2).

**Table 2:** Baseline characteristics for the patients included

	All patients (*n* = 212)	CAD (*n* = 82)	Non-CAD (*n* = 129)	*P* value
Age, years, mean ± SD	58.82 ± 13.71	64.5 ± 12.2	55.3 ± 13.5	< 0.001^***^
Sex, male (%)	137 (64.4%)	58 (70.7%)	79 (61.2%)	0.1835
HTN, n (%)	81 (38.2%)	37 (45.1%)	44 (34.1%)	0.1335
DM, n (%)	52 (24.5%)	24 (29.3%)	28 (21.7%)	0.2452
Hyperlipidemia, n (%)	62 (29.2%)	27 (32.9%)	35 (27.1%)	0.3096
Myocardium bridge, n (%)	50 (23.6%)	14 (17.1%)	36 (27.9%)	0.0019^**^
Clinical complaints when referred for CCTA				
Chest pain	138 (65.1%)	52 (63.4%)	86 (66.2%)	0.6836
Syncope	8 (37.7%)	5 (6.1%)	3 (2.3%)	0.1585
Dyspnea	17 (8.0%)	9 (11.0%)	8 (6.2%)	0.2080
Palpitation	27 (12.7%)	11 (13.4%)	16 (12.3%)	0.0187^*^
Physical examination	22 (10.4%)	5 (6.1%)	17 (13.1%)	0.1046

*CAD* coronary artery disease, *HTN* hypertension, *DM* diabetes mellitus

^*^: *p* < 0.05; ^**^: *p* < 0.01; ^***^: *p* < 0.001

### High-risk anatomic features analysis

A total of 6 types of ACAOS were recognised. The subpulmonic type (*n* = 97, 53·3%) was the most common for R-ACAOS, followed by the parapulmonic type (*n* = 63, 34.6%) and suprapulmonic/interarterial type (*n* = 22, 12.1%), while the retroaortic type (*n* = 14, 48·3%) was the most common for L-ACAOS, followed by prepulmonic type (*n* = 8, 27.6%),) and suprapulmonic/interarterial type (*n* = 7, 24.1%) (Fig. 4c). R-ACAOS patients were further stratified according to the degree of stenosis (severe stenosis: ≥ 50% stenosis; mild stenosis: < 50% stenosis). Among the 182 R-ACAOS patients, younger patients tended to have a higher degree of severity when referred for coronary CTA for the very first time (mild vs severe: 62·1 vs 57·0, *P* = 0·0018). A significant difference was observed within the 2 groups for the HW ratio (*P* = 0·0294), length of the proximal narrowing vessel (*P* = 0·0013), angle at the take-off level (*P* = 0·0238), and the ratio with possible associated coronary arteries and the ascending aorta (*P* < 0·001). No significant difference was found in the ostium type or the route of the abnormal RCA. Linear regression results showed significant correlations between the stenosis severity and the angle at take-off level (r = 0·31, *P* < 0·001), HW ratio (r = 0·20, *P* < 0·001), and the length of the proximal stenosis (r = 0·24, *P* < 0·001). Regarding the course of the abnormal RCA, no significant difference in severity was observed among the separate groups (Table 3).

**Table 3:** CTA findings of the patients with R-ACAOS

	Stenosis < 50% (*n* = 90)	Stenosis ≥50% (*n* = 92)	*P* value
Age, years	62.1 ± 11.6	57.0 ± 13.6	0.0018^*^
CAD, n (%)	44 (48.9%)	29 (32.2%)	0.0251
Myocardium bridge, n (%)	19(21.1%)	23(25.6%)	0.6551
High-risk CCTA sign			
HW ratio	1.83 ± 0.55	2.04 ± 0.72	0.0294^**^
Length of narrowing (cm)	2.35 ± 1.27	2.74 ± 0.96	0.0013^**^
Angle at take-off (°)	41.4 ± 21.7	33.2 ± 17.4	0.0238^**^
Shared ostium, n (%)	60 (66.7%)	68 (73.9%)	0.364
Ratio with vessels			
Ascending Aorta	19.66 ± 6.97	32.59 ± 26.30	< 0.001^**^
Left Main	2.56 ± 0.74	3.99 ± 1.07	< 0.001^**^
Left Anterior Descending	1.90 ± 0.56	2.95 ± 0.96	< 0.001^**^
Left Circumflex	1.70 ± 0.51	2.66 ± 0.88	< 0.001^**^
Vessel routes, n (%)			0.6588^†^
Above the PV	9(10.0%)	13 (14.1%)	
Below the PV	81(90.0%)	79 (85.9%)	

HW ratio: height to width ratio on the view vertical to the proximal stenosis vessel; Above the PV: the route starts at a suprapulmonic level and courses above the pulmonary valve; Below the PV: the route starts at a parapulmonic or subpulmonic level and courses below the pulmonic valve

^*^ The difference was tested by Student-t test

^**^ The difference was tested by Wilcox test for the obvious outlies found on the Q-Q plot

^†^ The *P* value is for testing the difference of severity among three type of vessel routes

With regard to the accompanying coronary artery variations, the dominant coronary artery type prevalence for L-ACAOS were 71·8% (right dominant), 12·45% (left dominant) and 16·47% (balanced type) and those for R-ACAOS were 93·1% (right dominant), 3·45% (left dominant) and 3·45% (balanced type) (Fig. 4a). One R-ACAOS case had no left main branch and 2 L-ACAOS cases had no left circumflex (LCX). No hypoplastic coronary was found in all ACAOS while 4 (13·3%) cases were found to have a hypertrophic RCA in the L-ACAOS group. Overall, 23·6% of patients had a myocardial bridge and it was found in a significantly higher frequency in the non-CAD group than in the CAD group (27·9% vs 17·1%, *P* = 0·0019).

### Analysis of clinical events in non-CAD R-ACAOS patients

A mean follow-up of 27.8 ± 18.7 months was performed on 108 non-CAD R-ACAOS patients (11 patients were lost due to ineffectively recorded corresponding numbers). Fifty-four patients reported CV-related clinical and 44 (40.7%) patients reported typical angina symptoms, among which none was with ACS. Eight patients died (one with lung cancer and one with rectal cancer, and the remaining 6 from sudden, unknown causes) and four patients reported arrhythmia. A total of 8 were recorded as MACE, including the 6 SCDs and 2 patients who had malignant arrhythmia and underwent pacemaker implantation or catheter ablation (Additional file 1: Table S1). No patient underwent vascular reconstruction surgery during our follow-up.

**Fig. 5:**
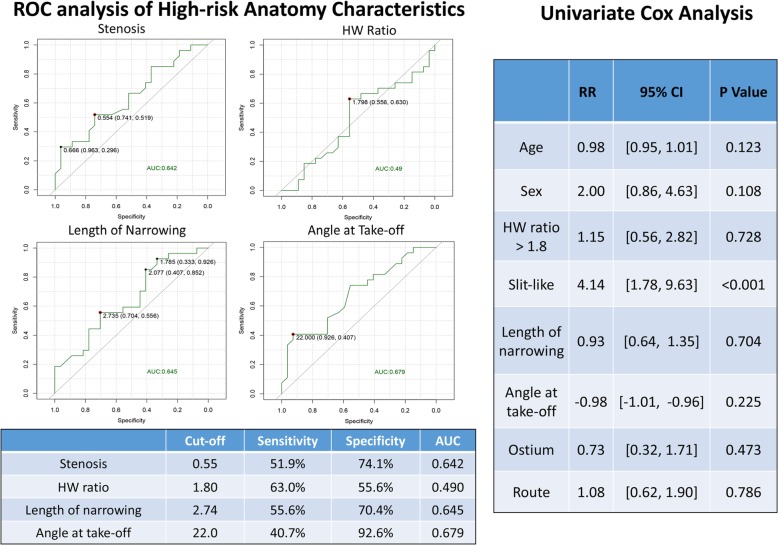
Receiver operating characteristics and Cox-univariate analysis for the prediction of MACE on the basis of high-risk anatomical characteristics. AUC: area under the curve; HW ratio: height-to-width ratio; HR: hazard ratio

No significant correlation was found between the frequency of chest pain and the occurrence of CV-related clinical events. Univariate regression revealed a significant correlation between the angle at the ostium (OR = − 0.04, STD = 0.02, *P* = 0.0196), the proximal stenosis length (OR = 0.67, STD = 0.32, *P* = 0.039), and the stenosis severity (OR = 3.87, STD = 1.87, *P* = 0.04). ROC analysis showed a moderate area under the curve with the stenosis severity (0.642, cutoff: 55%), length of narrowing (0·645, cutoff: 2.74 cm), and the angle at take-off level (0.679, cutoff: 22.0°). The Cox proportional hazards model revealed direct stenosis evaluation, including a slit-like shape, as the significant risk factor for CV-related clinical events. No significant differences were found in the HW ratio, take-off angle, proximal stenosis length, ostium type, and route type (Fig. 5).

## Discussion

The present study revealed that the prevalence of coronary CTA-detected ACAOS in our center, one of the highest-volume hospitals, was comparable to those of previous studies (0.69%). However, our patients exhibited a change of dominant coronary artery subsequent to ACAOS onset and generally smaller take-off angles. Moreover, in the present study, the incidence of ACAOS tended to be higher during the winter than in other seasons. In addition, we demonstrated that several high-risk morphological features recognized on coronary CTA might constitute risk factors for cardiovascular events in the mid-term follow-up, although stenosis severity was the only independent risk factor.

ACAOS-related SCD was first described by Cheitlin [30], after post-mortem examinations revealed an acute leftward passage between the aorta and the pulmonary trunk. Concerned with its potential to cause SCD, especially in the population with high myocardial load, an increasing number of studies have been conducted to find the high-risk anatomical factors of predictive value or to evaluate the safety and efficacy of surgical therapy. Compared with previous studies, our study took the lead in describing the prevalence and anatomical features of ACAOS on a large scale.

The prevalence of ACAOS and the ratio of R-ACAOS to L-ACAOS (182/29) were comparable to those of previous studies, and a noticeable regional difference in relation to data from the European area showed a higher prevalence of L-ACAOS, except a study by Kooij M [23] (Table 1). Major differences between ACAOS and normal patients were found, revealing a higher prevalence of both the left dominant coronary artery in the R-ACAOS group (12.5% vs 7–8%) and the right dominant coronary artery in the L-ACAOS group (93.1% vs 87–89%) [31], which, to our knowledge, has never been reported. Furthermore, a previous study showed a higher prevalence in young athletes [32]. In the present study, a lower age was observed in the group with higher severity.

Another interesting finding of our prevalence analysis was that the occurrence of ACAOS in patients referred to coronary CTA tended to be higher in the winter, which is in line with the reported seasonal tendency of coronary artery death. A possible reason for this finding may be that the cause of myocardial ischemia symptoms in ACAOS patients was similar to that of coronary artery death, as indicated in a previous study reporting that cold weather may place a higher demand in myocardial oxygen and blood pressure. Moreover, the higher rate of plasma lipids or respiratory diseases such as influenza might also play a role. This tendency may provide a clue taking seasonal changes into account when prescribing conservative therapy. However, further empirical epidemiology studies are required to confirm this fact.

Consistent with a previously reported SCD in R-ACAOS without necessary chest pain [22] or positive stress test results [33], in the present study, no significant correlation was found between chest pain complaint when referred to coronary CTA and the mid-term cardiovascular events. These results suggest that presence of chest pain may not be considered as the main risk factor for cardiovascular events, especially when handling plaque free ACAOS. However, when handling patients with evidence of atherosclerosis plaques, multiple factors may underlie the clinical manifestations and prognosis, making it difficult to attribute them arbitrarily to either the atherosclerotic plaques or anatomical anomaly. In our opinion, the systematic evaluation of the CAD, including the calcification score and stenosis severity according to the respective guidelines, as well as the downstream investigations including exercise testing, perfusion imaging, and catheter angiogram, et al. is a priority in CAD patients, whereas a meticulous review of the patients’ anatomical features is needed to exclude coronary anomaly, especially when handling patients with CAD symptoms of higher severity than those expected from the plaque itself. Recently, Driesen, et al. reported the value of fractional flow reserve (FFR) in better assessing the hemodynamic level of patients with ACAOS, which undoubtedly provided a new method to identify the patients at risk and helped with clinical decision making for patients with ACAOS [25].

Different theories have been proposed to explain the possible mechanism of SCD in ACAOS patients, corresponding to various high-risk anatomical features on coronary CTA [34]. Previously, L-ACAOS has been confirmed as a high-risk type and vascular reconstruction was recommended for such patients. In the present study, both the anatomical analysis and clinical outcomes provided mixed results for patients with R-ACAOS. Stenosis severity higher than 50%, designated as “slit-like” orifice, showed a promising predictive value for CV-related events (RR: 4.14, *P* < 0·001), which was consistent with previous studies. We then identified a smaller best threshold (angle < 22°) to define the “acute” take-off angle to predict CV-related clinical events in our group. Although this threshold has previously been defined as an angle < 30° [17] or 45° [22, 35], our study population showed a prominently smaller angle than the previous studies, with 92.3% exhibiting an angle < 45° and 84·6% presenting an angle < 30°, thus promoting a smaller threshold to clinically evaluate patients. Similarly, with the stenosis length, our ROC analysis showed a best threshold of 22 mm, which is nearly identical to that reported by Krupiński M [18], but much longer than those reported in other studies [24]. We believe that this measurement is related to the image reconstruction and to the operator, and an appropriate threshold length might need to be based more on the center’s own database.

Another anatomical feature considered as a potential “killer” is “intramural” [36], which was described by the theory arguing that the compression of the aorta or pulmonary trunk, or the outflow of the right ventricle, might cause transient stenosis of the abnormal CA’s affected segment, and the “intramural” route can add even higher and more direct pressure to the affected vessel segment [9]. Although most studies have used echocardiography to identify the occurrence of intramural artery, the definition of “intramural” requests histological confirmation of a shared media with the aorta and detached adventitia [31]. A study by Agrawal H et al. introduced a composite index to define the “intramural path,” based on a combination of a “pericoronary fat sign” and an “oval shape.” For the former, the “Fat sign” is relatively subjective, and our pre-test failed to achieve an adequate reproducibility when assessing the fat surrounding stenosis segments. The latter, which is also designated as HW ratio, has been recognized as a good assessment index. In the study by Miller JA [14], all patients exhibiting intramural artery route had an HW ratio higher than 1.5, and a mean of 2.19 was reported against 1.03 of the negative group. Another study [37] reported a discernible difference in the HW ratio according to whether the patients developed MACE or not. From the results of the present study, mixed consequences should be noted. First, an HW ratio higher than 1.8 was recognized as the best threshold to predict the mid-term cardiovascular events, although it failed to show a significant predictive value. Furthermore, a significantly different HW ratio was seen according to whether the severity of stenosis was lower or higher than 50%, with a significant correlation between the 2. Thus, the prognostic value of the HW found in the present study may reflect stenosis severity. Considering that previous studies have only included selective patients and that, although our study lacks surgery results, it is more general and population-based, it is safe to conclude that the higher degree of HW truly reflects the compression from nearby structures and possibly the presence of “intramural” variation, although by itself, it cannot determine the prognosis.

The take-off and course level of the aberrant coronary artery were also considered to influence the patients’ clinical outcome. Studies by Lee HJ et al. postulated a more prominent pressure from the pulmonic trunk than from the right ventricular outflow tract in the systole and proved a discernible higher rate of MACE in patients with supra-pulmonic RCA. However, the study by Ghoshhajra B [38] showed no significant difference on the take-off level height, taking the aortic commissure as the reference. Our results were consistent with those of the latter study.

## Limitations

Several limitations must be noted here: first, although based on a relatively large scale, the data in this study are from a single centre, and the patients from this study were major in middle aged group, thus possible bias still exists; Second, the follow-up results were from the telephone consultation and depended on the patients’ subjective feelings. And due to limited data on the exercise testing results or further investigations results for the patients complaining angina, we were not able to provide more accurate results for the associations between the patients’ symptoms with other clinical events. However, our patients were from a group taking up approximately 1/5 of the Chinese population, and outpatient follow-up for such big group was relatively difficult to perform. Third, because it was a retrospective study, all the clinical information was acquired from the database as it was recorded. We were not able to better rank or classify the chief complaints. Further study would be needed to confirm if exertional or non-exertional chest pain would be related to patients’ anatomy differences. Our evaluation of the coronary CTA images failed to have an impact on the physician’s management. Future prospective studies are needed to prove the clinical value of the anatomy factors for such patients. Forth, the biopsy data of the dead patients was not available, which make it impossible to conclude if the anatomy features of these patients contribute to the sudden death.

## Conclusion

The prevalence of ACAOS in our center was 0.69%, and our patients demonstrated a change of dominant coronary artery after ACAOS onset. In R-ACAOS patients with no coronary plaques, severe stenosis was the only risk factor for adverse prognosis, and thus, positive clinical intervention should at least be offered for such patients, as well as the patients with L-ACAOS. However, considering the significant anatomical variation among ACAOS patients, individual criteria to identify “high-risk” anatomy features should be developed to evaluate patients from different geographical regions.

## Supplementary information

**Additional file 1: Table S1.** CCTA findings and clinical information of the 8 patients with major adverse clinical events (MACE).

## Data Availability

The datasets generated and/or analysed during the current study are not publicly available due to the related rules of our hospital but are available from the corresponding author on reasonable request.

## Abbreviations

ACAOS: Anomalous origin of the coronary arteries from the opposite site
CAD: Coronary atherosclerosis
CTA: Computed tomography angiography
CTDI: CT dose index
DLP: Dose-length product
DSCT: Dual-source computed tomography
ED: Effective dose
HW ratio: Height-to-weight ratio
L-ACAOS: Abnormal left coronary artery from the right site
MACE: Major adverse clinical event
R-ACAOS ASACAOS: Abnormal right coronary artery from the left site
RCA: Right coronary artery
SCD: Sudden cardiac death
TEE: Transoesophageal echocardiography

## References

[1] Warnes CA, Williams RG, Bashore TM, Child JS, Connolly HM, Dearani JA (2008). ACC/AHA 2008 guidelines for the management of adults with congenital heart disease: a report of the American College of Cardiology/American Heart Association task force on practice guidelines. J Am Coll Cardiol.

[2] Eckart RE, Scoville SL, Campbell CL (2004). Sudden death in young adults : a 25-year review of autopsies in military recruits. Ann Intern Med.

[3] Maron BJ, Doerer JJ, Haas TS, Tierney DM, Mueller FO (2009). Sudden deaths in young competitive athletes analysis of 1866 deaths in the United States, 1980-2006. Circulation.

[4] Gaudin R, Raisky O, Vouhé PR (2014). Anomalous aortic origin of coronary arteries: ‘anatomical’ surgical repair. Multimed Man Cardiothorac Surg.

[5] Law T, Dunne B, Stamp N, Ho KM, Andrews D (2016). Surgical results and outcomes after Reimplantation for the Management of Anomalous Aortic Origin of the right coronary artery. Ann Thorac Surg.

[6] Feins EN, DeFaria YD, Bhatt AB, Stefanesce A, Youniss MA, Ghoshhajra BB (2016). Anomalous aortic origin of a coronary artery: surgical repair with anatomic- and function-based follow-up. Ann Thorac Surg.

[7] Shi K, Gao H, Yang Z, Zhang Q, Liu X, Guo Y (2017). Preoperative evaluation of coronary artery fistula using dual-source computed tomography. Int J Cardiol.

[8] Baumgartner H, Bonhoeffer P, De Groot NMS, De Haan F, Deanfield JE, Galie N (2010). ESC guidelines for the management of grown-up congenital heart disease (new version 2010). Eur Heart J.

[9] Angelini P, Velasco JA, Ott D, Khoshnevis GR (2003). Anomalous coronary artery arising from the opposite sinus: descriptive features and pathophysiologic mechanisms, as documented by intravascular ultrasonography. J Invasive Cardiol.

[10] Frommelt PC, Sheridan DC, Berger S, Frommelt MA, Tweddell JS (2011). Ten-year experience with surgical unroofing of anomalous aortic origin of a coronary artery from the opposite sinus with an interarterial course. J Thorac Cardiovasc Surg.

[11] Mumtaz MA, Lorber RE, Arruda J, Pettersson GB, Mavroudis C (2011). Surgery for anomalous aortic origin of the coronary artery. Ann Thorac Surg.

[12] Krasuski RA, Magyar D, Hart S, Kalahasti V, Lorber R, Hobbs R (2011). Long-term outcome and impact of surgery on adults with coronary arteries originating from the opposite coronary cusp. Circulation..

[13] Xu H, Zhu Y, Zhu X, Tang L, Xu Y (2012). Anomalous coronary arteries: depiction at dual-source computed tomographic coronary angiography. J Thorac Cardiovasc Surg.

[14] Miller JA, Anavekar NS, El Yaman MM, Burkhart HM, Miller AJ, Julsrud PR (2012). Computed tomographic angiography identification of intramural segments in anomalous coronary arteries with interarterial course. Int J Card Imaging.

[15] Lee H, Hong Y, Kim H, Lee L, Hur J, Choi B (2012). Anomalous origin of the right coronary artery from the left coronary sinus with an Interarterial course: subtypes and clinical importance. Radiology..

[16] Tuo G, Marasini M, Brunelli C, Zannini L, Balbi M (2013). Incidence and clinical relevance of primary congenital anomalies of the coronary arteries in children and adults. Cardiol Young.

[17] Opolski MP, Pregowski J, Kruk M, Witkowski A, Kwiecinska S, Lubienska E (2013). Prevalence and characteristics of coronary anomalies originating from the opposite sinus of valsalva in 8,522 patients referred for coronary computed tomography angiography. Am J Cardiol.

[18] Krupiński M, Urbańczyk-Zawadzka M, Laskowicz B, Irzyk M, Banyś R, Klimeczek P (2014). Anomalous origin of the coronary artery from the wrong coronary sinus evaluated with computed tomography: ‘high-risk’ anatomy and its clinical relevance. Eur Radiol.

[19] Sharma V, Burkhart HM, Dearani JA, Suri RM, Daly RC, Park SJ (2014). Surgical Unroofing of anomalous aortic origin of a coronary artery: a single-center experience. Ann Thorac Surg.

[20] Poynter JA, Bondarenko I, Austin EH, DeCampli WM, Jacobs JP, Ziemer G (2014). Repair of anomalous aortic origin of a coronary artery in 113 patients: a congenital heart surgeons’ society report. World J Pediatr Congenit Heart Surg.

[21] Ashrafpoor G, Danchin N, Houyel L, Ramadan R, Belli E, Paul JF (2014). Anatomical criteria of malignancy by computed tomography angiography in patients with anomalous coronary arteries with an interarterial course. Eur Radiol.

[22] Nasis A, Machado C, Cameron JD, Troupis JM, Meredith IT, Seneviratne SK (2014). Anatomic characteristics and outcome of adults with coronary arteries arising from an anomalous location detected with coronary computed tomography angiography. Int J Card Imaging.

[23] Kooij M, Vliegen HW, de Graaf MA, Hazekamp MG (2015). Surgical treatment of aberrant aortic origin of coronary arteries. Eur J Cardiothorac Surg.

[24] Cheezum Michael K., Ghoshhajra Brian, Bittencourt Marcio S., Hulten Edward A., Bhatt Ami, Mousavi Negareh, Shah Nishant R., Valente Anne Marie, Rybicki Frank J., Steigner Michael, Hainer Jon, MacGillivray Thomas, Hoffmann Udo, Abbara Suhny, Di Carli Marcelo F., DeFaria Yeh Doreen, Landzberg Michael, Liberthson Richard, Blankstein Ron (2016). Anomalous origin of the coronary artery arising from the opposite sinus: prevalence and outcomes in patients undergoing coronary CTA. European Heart Journal – Cardiovascular Imaging.

[25] Driesen BW, Warmerdam EG, Sieswerda GT (2018). Anomalous coronary artery originating from the opposite sinus of Valsalva (ACAOS), fractional flow reserve- and intravascular ultrasound-guided management in adult patients. Catheter Cardiovasc Interv.

[26] Cury RC, Abbara S, Achenbach S (2016). CAD-RADS(TM) coronary artery disease - reporting and data system. An expert consensus document of the Society of Cardiovascular Computed Tomography (SCCT), the American College of Radiology (ACR) and the north American Society for Cardiovascular Imaging (NASCI). Endorsed by the American College of Cardiology. J Cardiovasc Comput Tomogr.

[27] Virmani R, Chun PKC, Goldstein RE (1984). Acute takeoffs of the coronary arteries along the aortic wall and congenital coronary ostial valve-like ridges: association with sudden death. J Am Coll Cardiol.

[28] Agrawal H, Mery CM, Krishnamurthy R, Molossi S (2017). Anatomic types of anomalous aortic origin of a coronary artery: a pictorial summary. Congenit Heart Dis.

[29] Deak PD, Smal Y, Kalender WA (2010). Multisection CT Protocols: Sex- and Age-specific Conversion Factor used to determine Effective Dose from Dose-Length Product. Radiology..

[30] Cheitlin MD, De Castro CM, Mcallister HA (1974). Sudden Death as a Complication of Anomalous Left Coronary Origin From the Anterior Sinus of Valsalva. Circulation.

[31] Cademartiri F, La Grutta L, Malago R, Alberghina F, Meijboom WB, Pugliese F (2008). Prevalence of anatomical variants and coronary anomalies in 543 consecutive patients studied with 64-slice CT coronary angiography. Eur Radiol.

[32] Basso C, Maron BJ, Corrado D, Thiene G (2000). Clinical profile of congenital coronary artery anomalies with origin from the wrong aortic sinus leading to sudden death in young competitive athletes. J Am Coll Cardiol.

[33] Jo Y, Uranaka Y, Iwaki H, Matsumoto J, Koura T, Negishi K (2011). Sudden cardiac arrest: associated with anomalous origin of the right coronary artery from the left main coronary artery. Tex Heart Inst J.

[34] McLarry J, Ferencik M, Shapiro MD (2015). Coronary artery anomalies: a pictorial review. Curr Cardiovasc Imaging Rep.

[35] Brothers JA, Gaynor JW, Jacobs JP, Caldarone C, Jegatheeswaran A (2010). The registry of anomalous aortic origin of the coronary artery of the congenital heart surgeons’ society. Cardiol Young.

[36] Kaushal S, Backer CL, Popescu AR, Walker BL, Hyde M, Koenig PR (2011). Intramural coronary length correlates with symptoms in patients with anomalous aortic origin of the coronary artery. Ann Thorac Surg.

[37] Abbara S, Arbab-Zadeh A, Callister TQ, Desai MY, Mamuya W, Thomson L (2009). SCCT guidelines for performance of coronary computed tomographic angiography: a report of the Society of Cardiovascular Computed Tomography Guidelines Committee. J Cardiovasc Comput Tomogr.

[38] Lee Bae Young, Song Kyung Sup, Jung Seung Eun, Jung Jung Im, Chun Ho Jong, Park Chan Beom, Kim Chi Kyung, Cho Eun Ju, Jin Ung, Jung Hae Ok (2009). Anomalous Right Coronary Artery Originated From Left Coronary Sinus With Interarterial Course. Journal of Computer Assisted Tomography.

