# Supplementary material for: Prognostic value of dual-source computed tomography (DSCT) angiography characteristics in anomalous coronary artery from the opposite sinus (ACAOS) patients: a large-scale retrospective study
Source: BMC Cardiovasc Disord. 2020 Jan 17;20:25. doi: 10.1186/s12872-019-01285-3 (PMC6966895; doi:10.1186/s12872-019-01285-3)
Supplement: Supplementary file 1 — Additional file 1: Table S1. CCTA findings and clinical information of the 8 patients with major adverse clinical events (MACE). [file 12872_2019_1285_MOESM1_ESM.docx]

| Patient No | Reasons for CCTA | Route | Shape | HW ratio | Take-off angle | Typical Angina symptoms | MACE |
| --- | --- | --- | --- | --- | --- | --- | --- |
| 1 | Chest pain | subpulmonic | Slit-like | 3 | 15 | F | death |
| 2 | Chest pain | parapulmonic | Slit-like | 5.64 | 25 | F | death |
| 3 | syncope | subpulmonic | Slit-like | 2.76 | 9 | F | death |
| 4 | Chest pain | parapulmonic | Slit-like | 2.85 | 16 | T | death |
| 5 | Chest pain | suprapulmonic | oval | 1.81 | 16 | T | death |
| 6 | Chest pain | parapulmonic | Slit-like | 2.36 | 19 | T | death |
| 7 | Chest pain | subpulmonic | oval | 1.58 | 5 | T | VT |
| 8 | Chest pain | parapulmonic | Slit-like | 2.71 | 28 | T | VF |

Table S1. CCTA findings and clinical information of the 8 patients with major adverse clinical events (MACE). H-W ratio: height-to-weight ratio; The shape is considered round with a HW ratio less than 1.11; oval with a H-W ratio between 1.11 and 2 ; slit-like with a H-W ratio more than 2^1^. ; VT: ventricular tachycardia; VF: ventricular fibrillation.

Reference:

1. *Agrawal H, Mery CM, Krishnamurthy R, Molossi S. Anatomic types of anomalous aortic origin of a coronary artery: A pictorial summary. Congenital Heart Disease. 2017;00:1-4*)
